# Di-μ-azido-di­azidodi-μ-oxalato-di­histamine­tetra­copper(II) 0.9-hydrate

**DOI:** 10.1107/S1600536813013329

**Published:** 2013-05-22

**Authors:** Chen Liu, Khalil A. Abboud

**Affiliations:** a Department of Chemistry and Environmental Science, Grenfell Campus, Memorial University of Newfoundland, Corner Brook, Newfoundland, A2H 6P9, Canada; b Department of Chemistry, University of Florida, Gainesville, FL 32611-7200, USA

## Abstract

The title compound, [Cu_4_(C_2_O_4_)_2_(N_3_)_4_(C_5_H_9_N_3_)_2_]·0.9H_2_O, contains a tetranuclear Cu^II^-based molecule composed of two oxalate-bridged Cu^II^ dimers linked through end-on azide ions and related by an inversion center. The tetranuclear unit contains two crystallographically independent Cu^II^ ions. One Cu^II^ ion coordinates to two N atoms of a histamine mol­ecule, two O atoms of a bridging oxalate ligand, and an N atom of an end-on bridging azide ligand, leading to an elongated square-pyramidal coordination geometry in which the azide ion occupies the axial position. The other Cu^II^ ion, which has a square-planar coordination geometry, is coordinated by two O atoms of a bridging oxalate ligand and two N atoms of two different azide ligands, one which is bridging. In the crystal, a two-dimensional network parallel to (010) is formed by N—H⋯N and N—H⋯O hydrogen bonds. A partially occupied solvent water mol­ecule refined to an occupancy of 0.447 (5). Two of the azide ligands were refined as disordered over two sets of sites with refined occupancies in the ratios 0.517 (8):0.483 (8) and 0.553 (5):0.447 (5).

## Related literature

For background to bridging oxalate and azide ligands, see: Coronado *et al.* (2003 ▶); Ribas *et al.* (1999 ▶); Pardo *et al.* (2010 ▶); Sun *et al.* (1997 ▶). 
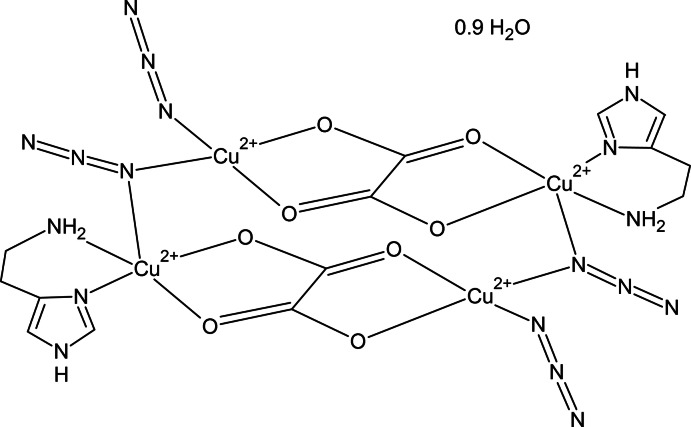


## Experimental

### 

#### Crystal data

[Cu_4_(C_2_O_4_)_2_(N_3_)_4_(C_5_H_9_N_3_)_2_]·0.9H_2_O
*M* *_r_* = 838.62
Triclinic, 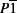

*a* = 7.7003 (10) Å
*b* = 8.2841 (11) Å
*c* = 11.8677 (15) Å
α = 106.005 (2)°
β = 91.715 (2)°
γ = 115.010 (2)°
*V* = 650.07 (15) Å^3^
*Z* = 1
Mo *K*α radiation
μ = 3.31 mm^−1^
*T* = 173 K
0.12 × 0.11 × 0.04 mm

#### Data collection

Bruker SMART CCD area-detector diffractometer
Absorption correction: integration [based on measured indexed crystal faces (*SHELXTL*; Sheldrick, 2008 ▶)] *T*_min_ = 0.654, *T*_max_ = 0.877
5770 measured reflections
2885 independent reflections
2442 reflections with *I* > 2σ(*I*)
*R*_int_ = 0.041

#### Refinement

*R*[*F* ^2^ > 2σ(*F* ^2^)] = 0.029
*wR*(*F* ^2^) = 0.073
*S* = 1.03
2885 reflections
238 parameters
H atoms treated by a mixture of independent and constrained refinement
Δρ_max_ = 0.60 e Å^−3^
Δρ_min_ = −0.56 e Å^−3^

### 

Data collection: *APEX2* (Bruker, 2008 ▶); cell refinement: *SAINT* (Bruker, 2008 ▶); data reduction: *SAINT*; program(s) used to solve structure: *SHELXTL* (Sheldrick, 2008 ▶); program(s) used to refine structure: *SHELXTL*; molecular graphics: *SHELXTL*; software used to prepare material for publication: *SHELXTL*.

## Supplementary Material

Crystal structure: contains datablock(s) I, global. DOI: 10.1107/S1600536813013329/lh5597sup1.cif

Structure factors: contains datablock(s) I. DOI: 10.1107/S1600536813013329/lh5597Isup2.hkl

Additional supplementary materials:  crystallographic information; 3D view; checkCIF report

## Figures and Tables

**Table 1:** Hydrogen-bond geometry (Å, °)

*D*—H⋯*A*	*D*—H	H⋯*A*	*D*⋯*A*	*D*—H⋯*A*
N9—H9⋯O5^i^	0.80 (4)	2.18 (4)	2.861 (6)	143 (3)
N9—H9⋯N6′^i^	0.80 (4)	2.59 (4)	3.171 (6)	130 (3)
N9—H9⋯N3′^ii^	0.80 (4)	2.60 (4)	3.145 (7)	127 (3)
N9—H9⋯N6^iii^	0.80 (4)	2.38 (4)	3.108 (6)	151 (3)

Symmetry codes: (i) 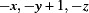; (ii) 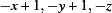; (iii) 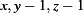.

**Fig. 1.**
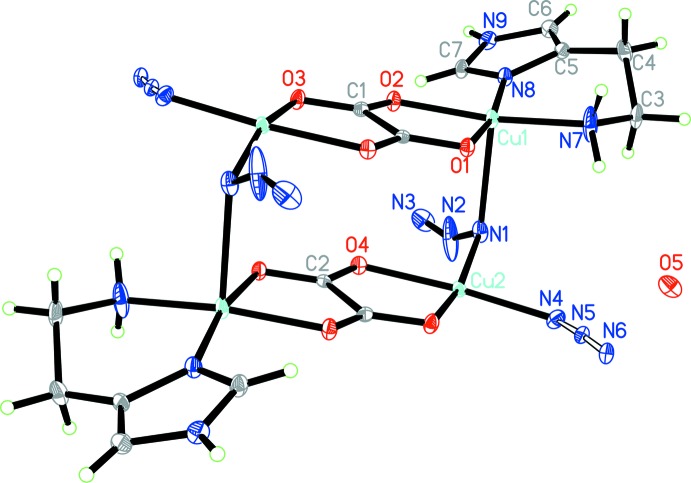
The molecular structure of the title compound, with ellipsoids drawn at 30% probability level. Unlabelled atoms are related by the symmetry transofrmation: -x + 1, -y + 1, -z + 1.

**Fig. 2.**
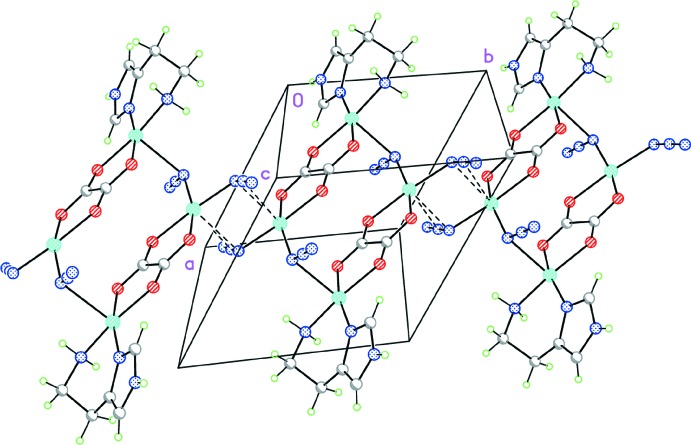
One-dimensional chain of tetramers of the title compound.

## supplementary crystallographic information

### Comment

The title compound is a polynuclear coordination complex involving mixed bridging ligands, both oxalate (Coronado *et al.* 2003; Pardo *et al.* 2010; Sun *et al.*, 1997) and azide (Ribas, *et al.* 1999) anions. The tetrameric unit in the title compound is centrosymmetric (Fig.1), so pairs of equivalent ligands lie *trans* to each other. Two of the four azide ions within each tetramer are in end-on bridging mode, while the other two are non-bridging but form long N4A–Cu[1-x, 2-y, 1-z] (two-dimensional) bonds of 2.486 (3) Å with Cu^II^ ions on neighbouring tetramers and therefore link tetramers along the crystallographic *b* axis to produce one-dimensional chains (Fig.2). The distance between the two Cu^II^ ions linked by this long Cu—N bond is 3.2261 (8) Å.

### Experimental

An aqueous solution (15 ml) of copper(II) nitrate trihydrate (4.0 mmol, 0.97 g) was slowly added to an aqueous solution (50 ml) containing histamine dihydrochloride (4.0 mmol, 0.74 g), sodium oxalate (2.0 mmol, 0.27 g), sodium azide (4.0 mmol, 0.26 g), and sodium hydroxide (8.0 mmol, 0.32 g). The mixture was stirred for 10 minutes and allowed to stand in air. Green platelet crystals were collected after a few days and washed with deionized water and dried in air.

### Refinement

All H atoms were positioned geometrically (C—H = 0.93/1.00 Å) and allowed to ride with *U*_iso_(H)= 1.2/1.5*U*_eq_(C). Methyl H atoms were allowed to rotate around the corresponding C—C.

The N2—N3 and N5—N6 moieties were refined a disordered and each refined in two parts against N2'-N3' and N5'-N6'. A partial water molecule alternates with the N5'-N6' moiety in occupying the same space. The two N9 protons were obtained from a difference Fourier map but did not refine properly thus they were refined in idealized positions and were riding on their parent atom. The N9 proton is refined freely. All disordered parts have properly refined occupancy factors.

### Crystal data

**Table:**

[Cu_4_(C_2_O_4_)_2_(N_3_)_4_(C_5_H_9_N_3_)_2_]·0.9H_2_O	*Z* = 1
*M**_r_* = 838.62	*F*(000) = 416
Triclinic, *P*1	*D*_x_ = 2.137 Mg m^−^^3^
Hall symbol: -P 1	Mo *K*α radiation, λ = 0.71073 Å
*a* = 7.7003 (10) Å	Cell parameters from 29 reflections
*b* = 8.2841 (11) Å	θ = 2.0–28.0°
*c* = 11.8677 (15) Å	µ = 3.31 mm^−^^1^
α = 106.005 (2)°	*T* = 173 K
β = 91.715 (2)°	Plate, green
γ = 115.010 (2)°	0.12 × 0.11 × 0.04 mm
*V* = 650.07 (15) Å^3^	

### Data collection

**Table:**

Bruker SMART CCD area-detector diffractometer	2885 independent reflections
Radiation source: fine-focus sealed tube	2442 reflections with *I* > 2σ(*I*)
Graphite monochromator	*R*_int_ = 0.041
ω and φ scans	θ_max_ = 27.5°, θ_min_ = 1.8°
Absorption correction: integration [based on measured indexed crystal faces (*SHELXTL*; Sheldrick, 2008)]	*h* = −9→9
*T*_min_ = 0.654, *T*_max_ = 0.877	*k* = −10→10
5770 measured reflections	*l* = −15→15

### Refinement

**Table:**

Refinement on *F*^2^	Primary atom site location: structure-invariant direct methods
Least-squares matrix: full	Secondary atom site location: difference Fourier map
*R*[*F*^2^ > 2σ(*F*^2^)] = 0.029	Hydrogen site location: inferred from neighbouring sites
*wR*(*F*^2^) = 0.073	H atoms treated by a mixture of independent and constrained refinement
*S* = 1.03	*w* = 1/[σ^2^(*F*_o_^2^) + (0.0327*P*)^2^ + 0.5471*P*] where *P* = (*F*_o_^2^ + 2*F*_c_^2^)/3
2885 reflections	(Δ/σ)_max_ < 0.001
238 parameters	Δρ_max_ = 0.60 e Å^−^^3^
0 restraints	Δρ_min_ = −0.56 e Å^−^^3^

### Special details

**Table:**

Geometry. All e.s.d.'s (except the e.s.d. in the dihedral angle between two l.s. planes) are estimated using the full covariance matrix. The cell e.s.d.'s are taken into account individually in the estimation of e.s.d.'s in distances, angles and torsion angles; correlations between e.s.d.'s in cell parameters are only used when they are defined by crystal symmetry. An approximate (isotropic) treatment of cell e.s.d.'s is used for estimating e.s.d.'s involving l.s. planes.

### Fractional atomic coordinates and isotropic or equivalent isotropic displacement parameters (Å^2^)

**Table:**

	*x*	*y*	*z*	*U*_iso_*/*U*_eq_	Occ. (<1)
Cu1	0.10361 (5)	0.35821 (5)	0.26714 (3)	0.0187 (1)	
Cu2	0.45210 (4)	0.78190 (4)	0.47013 (3)	0.0146 (1)	
O1	0.1735 (3)	0.3957 (2)	0.44160 (16)	0.0177 (4)	
O2	0.2983 (3)	0.2567 (3)	0.24786 (16)	0.0196 (4)	
O3	0.4798 (3)	0.1874 (3)	0.35955 (16)	0.0194 (4)	
O4	0.3611 (3)	0.3319 (3)	0.55217 (16)	0.0172 (4)	
O5	−0.0013 (8)	0.9226 (8)	0.3473 (5)	0.0304 (14)	0.447 (5)
N1	0.3411 (3)	0.6717 (3)	0.3003 (2)	0.0217 (5)	
N2	0.4238 (7)	0.6559 (8)	0.2176 (4)	0.0181 (11)*	0.517 (8)
N3	0.5044 (9)	0.6493 (9)	0.1379 (5)	0.0389 (17)*	0.517 (8)
N2'	0.4711 (8)	0.7391 (9)	0.2413 (5)	0.0189 (12)*	0.483 (8)
N3'	0.5775 (9)	0.7833 (9)	0.1768 (6)	0.0378 (18)*	0.483 (8)
N4	0.2809 (4)	0.9047 (3)	0.5128 (2)	0.0218 (5)	
N5'	0.1670 (6)	0.8882 (5)	0.4349 (3)	0.0153 (10)	0.553 (5)
N6'	0.0499 (8)	0.8764 (8)	0.3687 (5)	0.0245 (11)	0.553 (5)
N5	0.2251 (7)	0.9251 (7)	0.6159 (4)	0.0182 (12)	0.447 (5)
N6	0.1813 (7)	0.9522 (7)	0.7129 (5)	0.0228 (13)	0.447 (5)
N7	−0.1142 (4)	0.4201 (5)	0.3030 (2)	0.0455 (9)	
H7B	−0.2026	0.3281	0.3293	0.055*	
H7C	−0.0665	0.5310	0.3651	0.055*	
H7A	0.245 (4)	0.240 (4)	0.028 (3)	0.016 (7)*	
N8	0.0241 (3)	0.2683 (3)	0.09526 (19)	0.0183 (5)	
N9	0.0241 (4)	0.1696 (3)	−0.0948 (2)	0.0215 (5)	
H9	0.064 (5)	0.142 (5)	−0.155 (3)	0.036 (10)*	
C1	0.3613 (4)	0.2501 (3)	0.3439 (2)	0.0163 (5)	
C2	0.2908 (3)	0.3308 (3)	0.4554 (2)	0.0137 (5)	
C3	−0.2212 (4)	0.4403 (5)	0.2049 (3)	0.0296 (7)	
H3B	−0.1315	0.5458	0.1795	0.035*	
H3A	−0.3275	0.4689	0.2337	0.035*	
C4	−0.3049 (4)	0.2605 (4)	0.1001 (2)	0.0258 (6)	
H4B	−0.3748	0.1526	0.1291	0.031*	
H4A	−0.4004	0.2647	0.0446	0.031*	
C5	−0.1530 (4)	0.2306 (4)	0.0346 (2)	0.0186 (5)	
C6	−0.1515 (4)	0.1704 (4)	−0.0830 (3)	0.0226 (6)	
H6A	−0.237 (5)	0.131 (5)	−0.143 (3)	0.027 (9)*	
C7	0.1261 (4)	0.2291 (4)	0.0137 (2)	0.0205 (6)	

### Atomic displacement parameters (Å^2^)

**Table:**

	*U*^11^	*U*^22^	*U*^33^	*U*^12^	*U*^13^	*U*^23^
Cu1	0.01983 (18)	0.02819 (19)	0.01006 (17)	0.01687 (15)	−0.00150 (12)	−0.00048 (13)
Cu2	0.01364 (16)	0.01637 (16)	0.01463 (17)	0.00709 (13)	0.00059 (12)	0.00577 (12)
O1	0.0189 (9)	0.0221 (9)	0.0142 (9)	0.0132 (8)	0.0004 (7)	0.0024 (7)
O2	0.0227 (10)	0.0281 (10)	0.0110 (9)	0.0176 (8)	−0.0011 (7)	0.0010 (7)
O3	0.0239 (10)	0.0238 (9)	0.0135 (9)	0.0169 (8)	−0.0001 (7)	0.0004 (8)
O4	0.0188 (9)	0.0212 (9)	0.0140 (9)	0.0108 (8)	0.0038 (7)	0.0058 (7)
O5	0.026 (3)	0.041 (3)	0.014 (2)	0.009 (2)	0.003 (2)	0.003 (2)
N1	0.0218 (12)	0.0337 (13)	0.0138 (11)	0.0166 (10)	0.0024 (9)	0.0071 (10)
N4	0.0303 (13)	0.0213 (11)	0.0163 (11)	0.0162 (10)	−0.0019 (10)	0.0030 (9)
N5'	0.017 (2)	0.0134 (18)	0.014 (2)	0.0076 (16)	0.0051 (16)	0.0018 (15)
N6'	0.020 (3)	0.034 (3)	0.023 (3)	0.015 (2)	−0.001 (2)	0.010 (2)
N5	0.018 (3)	0.018 (2)	0.023 (3)	0.012 (2)	0.004 (2)	0.007 (2)
N6	0.019 (3)	0.025 (3)	0.029 (3)	0.015 (2)	0.006 (2)	0.007 (2)
N7	0.0454 (17)	0.089 (2)	0.0143 (13)	0.0549 (18)	−0.0038 (12)	−0.0055 (14)
N8	0.0199 (11)	0.0245 (11)	0.0121 (10)	0.013 (1)	0.0009 (8)	0.0034 (9)
N9	0.0298 (13)	0.0263 (12)	0.0104 (11)	0.0157 (11)	0.004 (1)	0.0037 (10)
C1	0.0168 (12)	0.0147 (11)	0.0140 (12)	0.0068 (10)	−0.0002 (10)	0.0003 (10)
C2	0.0131 (12)	0.0117 (11)	0.0126 (12)	0.0042 (9)	−0.0007 (9)	0.0011 (9)
C3	0.0287 (16)	0.0449 (18)	0.0234 (15)	0.0297 (15)	0.0015 (12)	0.0019 (13)
C4	0.0208 (14)	0.0399 (16)	0.0176 (14)	0.0168 (13)	0.0009 (11)	0.0053 (12)
C5	0.0194 (13)	0.0202 (12)	0.0143 (12)	0.0104 (11)	−0.0042 (10)	0.0011 (10)
C6	0.0252 (15)	0.0252 (14)	0.0151 (13)	0.0127 (12)	−0.0038 (11)	0.0017 (11)
C7	0.0220 (14)	0.0272 (14)	0.0155 (13)	0.0164 (12)	0.0034 (11)	0.0029 (11)

### Geometric parameters (Å, º)

**Table:**

Cu1—N8	1.947 (2)	N5'—N6'	1.132 (7)
Cu1—N7	1.977 (2)	N5—N6	1.197 (7)
Cu1—O2	1.9970 (18)	N7—C3	1.493 (4)
Cu1—O1	2.0275 (18)	N7—H7B	0.9200
Cu1—N1	2.371 (3)	N7—H7C	0.9200
Cu2—N1	1.962 (2)	N8—C7	1.322 (3)
Cu2—N4	1.980 (2)	N8—C5	1.390 (3)
Cu2—O3^i^	1.9915 (18)	N9—C7	1.334 (4)
Cu2—O4^i^	2.0148 (18)	N9—C6	1.366 (4)
O1—C2	1.258 (3)	N9—H9	0.80 (4)
O2—C1	1.249 (3)	C1—C2	1.534 (3)
O3—C1	1.258 (3)	C3—C4	1.516 (4)
O3—Cu2^i^	1.9915 (18)	C3—H3B	0.9900
O4—C2	1.251 (3)	C3—H3A	0.9900
O4—Cu2^i^	2.0148 (18)	C4—C5	1.493 (4)
N1—N2	1.192 (5)	C4—H4B	0.9900
N1—N2'	1.260 (6)	C4—H4A	0.9900
N2—N3	1.149 (7)	C5—C6	1.348 (4)
N2'—N3'	1.153 (7)	C6—H6A	0.84 (3)
N4—N5'	1.192 (4)	C7—H7A	0.88 (3)
N4—N5	1.300 (5)		
			
N8—Cu1—N7	95.03 (10)	Cu1—N7—H7C	107.9
N8—Cu1—O2	90.05 (8)	H7B—N7—H7C	107.2
N7—Cu1—O2	168.22 (12)	C7—N8—C5	106.7 (2)
N8—Cu1—O1	168.11 (8)	C7—N8—Cu1	126.94 (19)
N7—Cu1—O1	89.88 (9)	C5—N8—Cu1	126.40 (18)
O2—Cu1—O1	83.23 (7)	C7—N9—C6	108.3 (2)
N8—Cu1—N1	101.85 (9)	C7—N9—H9	124 (3)
N7—Cu1—N1	95.97 (12)	C6—N9—H9	127 (3)
O2—Cu1—N1	93.37 (8)	O2—C1—O3	126.8 (2)
O1—Cu1—N1	88.36 (8)	O2—C1—C2	117.0 (2)
N1—Cu2—N4	94.99 (9)	O3—C1—C2	116.2 (2)
N1—Cu2—O3^i^	162.53 (9)	O4—C2—O1	126.1 (2)
N4—Cu2—O3^i^	90.34 (8)	O4—C2—C1	116.7 (2)
N1—Cu2—O4^i^	91.90 (8)	O1—C2—C1	117.1 (2)
N4—Cu2—O4^i^	173.09 (8)	N7—C3—C4	110.3 (3)
O3^i^—Cu2—O4^i^	82.91 (7)	N7—C3—H3B	109.6
C2—O1—Cu1	110.59 (16)	C4—C3—H3B	109.6
C1—O2—Cu1	111.93 (17)	N7—C3—H3A	109.6
C1—O3—Cu2^i^	112.47 (16)	C4—C3—H3A	109.6
C2—O4—Cu2^i^	111.66 (16)	H3B—C3—H3A	108.1
N2—N1—Cu2	128.1 (3)	C5—C4—C3	112.7 (2)
N2'—N1—Cu2	109.1 (3)	C5—C4—H4B	109.0
N2—N1—Cu1	102.3 (3)	C3—C4—H4B	109.0
N2'—N1—Cu1	130.6 (3)	C5—C4—H4A	109.0
Cu2—N1—Cu1	107.77 (10)	C3—C4—H4A	109.0
N3—N2—N1	176.5 (6)	H4B—C4—H4A	107.8
N3'—N2'—N1	172.4 (7)	C6—C5—N8	108.1 (2)
N5'—N4—N5	114.0 (3)	C6—C5—C4	130.8 (2)
N5'—N4—Cu2	117.9 (2)	N8—C5—C4	121.0 (2)
N5—N4—Cu2	121.0 (3)	C5—C6—N9	106.8 (2)
N6'—N5'—N4	173.9 (5)	C5—C6—H6A	132 (2)
N6—N5—N4	176.8 (5)	N9—C6—H6A	121 (2)
C3—N7—Cu1	117.6 (2)	N8—C7—N9	110.1 (2)
C3—N7—H7B	107.9	N8—C7—H7A	125.6 (19)
Cu1—N7—H7B	107.9	N9—C7—H7A	124.4 (19)
C3—N7—H7C	107.9		

Symmetry code: (i) −*x*+1, −*y*+1, −*z*+1.

### Hydrogen-bond geometry (Å, º)

**Table:**

*D*—H···*A*	*D*—H	H···*A*	*D*···*A*	*D*—H···*A*
N9—H9···O5^ii^	0.80 (4)	2.18 (4)	2.861 (6)	143 (3)
N9—H9···N6′^ii^	0.80 (4)	2.59 (4)	3.171 (6)	130 (3)
N9—H9···N3′^iii^	0.80 (4)	2.60 (4)	3.145 (7)	127 (3)
N9—H9···N6^iv^	0.80 (4)	2.38 (4)	3.108 (6)	151 (3)

Symmetry codes: (ii) −*x*, −*y*+1, −*z*; (iii) −*x*+1, −*y*+1, −*z*; (iv) *x*, *y*−1, *z*−1.
